# Towards universal health coverage in the context of population ageing: a narrative review on the implications from the long-term care system in Japan

**DOI:** 10.1186/s13690-022-00970-8

**Published:** 2022-09-21

**Authors:** Shohei Okamoto, Kohei Komamura

**Affiliations:** 1 Research Team for Social Participation and Community Health, Tokyo Metropolitan Institute of Gerontology, 35-2 Sakae-cho, Itabashi-ku, Tokyo, 1730015 Japan; 2 National Center for Global Health and Medicine, Institute for Global Health Policy Research, Tokyo, Japan; 3 Research Center for Financial Gerontology, Keio University, Tokyo, Japan; 4 Faculty of Economics, Keio University, Tokyo, Japan

**Keywords:** Long-term care, Universal health coverage, Population ageing, Healthy ageing, Japan

## Abstract

The two important elements of universal health coverage—(1) enabling everyone to access the necessary health services and (2) providing financial protection from catastrophic health spending—are vital for not only healthcare but also long-term care in the context of population ageing. In this review, we provide an overview of the public long-term care system in Japan to help other countries that are experiencing (or are expected to experience) problems associated with population ageing. Japan’s approach to long-term care may not be universally generalisable, given the differences in population/geographical sizes, socioeconomic development, population ageing, and cultures across countries. However, the challenges faced by older people may be common. Japan’s long-term care system has several challenges, including financing, labour force shortages, support for people with dementia, an integrated continuum of healthcare and long-term care, and utilising services outside the purview of insurance coverage. We have provided the government’s actions and potential directions to address these challenges.

Achieving universal health coverage (UHC) amidst population ageing and supporting the health and lives of older people is indispensable. Globally, all countries are either experiencing or are expected to experience rapid population ageing. Due to these changes, the progress towards UHC may decelerate because older people tend to need care for multiple chronic conditions, long-term care, and community support [1–6], which is not well considered in the current UHC scheme [7], and may need more care increasing associated financial burden. Thus, the framework of UHC needs to be reconsidered in the context of population ageing. As one of the “global leaders” in population ageing, Japan’s experiences in health and long-term care (LTC) policies for older people can be an asset to other countries. Therefore, we provide an overview of Japan’s LTC policy, which can serve as an example for other countries in developing care systems for their older population.

## Population ageing in Japan and the Western Pacific region

**Fig. 1:**
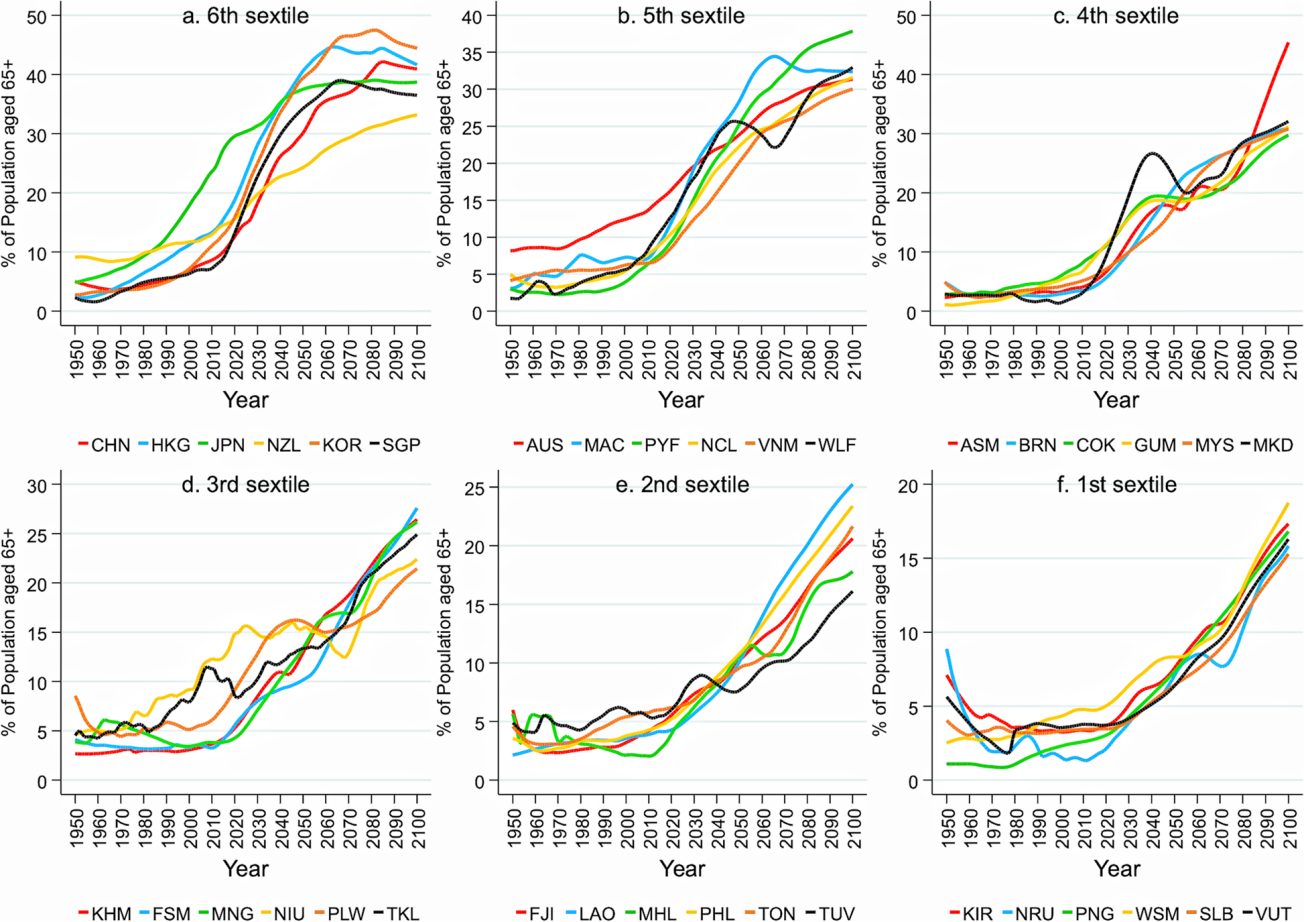
Actual and projected trends of ageing rate in the WHO Western Pacific region. Note: The ageing rate denotes the percentage of people age 65 or older among the total population; Countries included here are American Samoa (ASM), Australia (AUS), Brunei Darussalam (BRN), Cambodia (KHM), China (CHN), Hong Kong SAR (HKG), Macao SAR (MAC), Cook Islands (COK), Fiji (FJI), French Polynesia (PYF), Guam (GUM), Japan (JPN), Kiribati (KIR), Lao People’s Democratic Republic (LAO), Malaysia (MYS), Marshall Islands (MHL), The Federated States of Micronesia (FSM), Mongolia (MNG), Nauru (NRU), New Caledonia (NCL), New Zealand (NZL), Niue (NIU), Northern Mariana Islands (MKD), Palau (PLW), Papua New Guinea (PNG), Philippines (PHL), Republic of Korea (KOR), Samoa (WSM), Singapore (SGP), Solomon Islands (SLB), Tokelau (TKL), Tonga (TON), Tuvalu (TUV), Vanuatu (VUT), Viet Nam (VNM), and Wallis and Futuna (WLF). The data were obtained from the World Population Prospects 2022 [8]

**Fig. 2:**
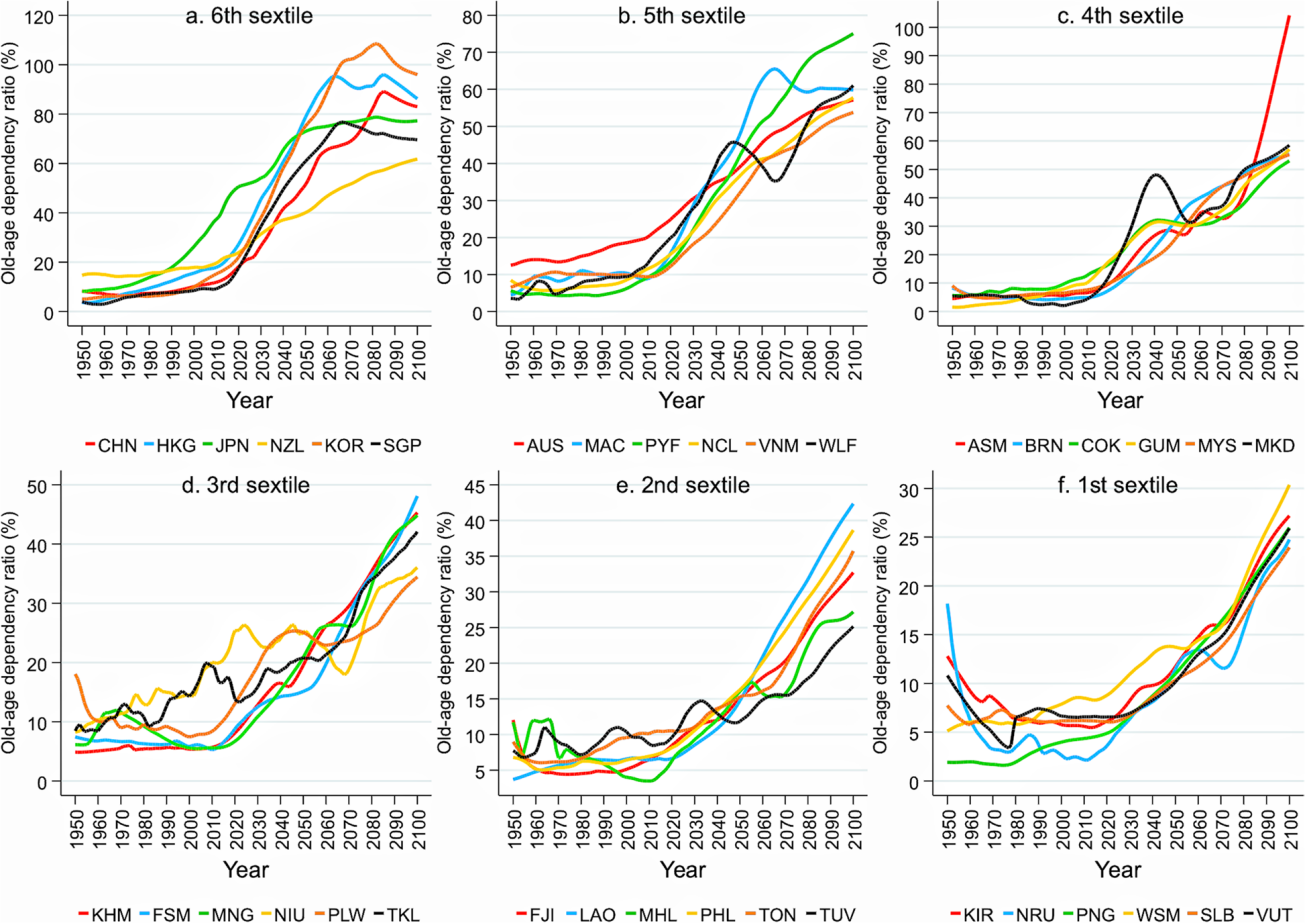
Actual and projected trends of old-age dependency ratio in the WHO Western Pacific region. Note: Old-age dependency ratio denotes the ratio between the population aged 65 or over and the working-age populations (15–64-year-olds); Countries included here are American Samoa (ASM), Australia (AUS), Brunei Darussalam (BRN), Cambodia (KHM), China (CHN), Hong Kong SAR (HKG), Macao SAR (MAC), Cook Islands (COK), Fiji (FJI), French Polynesia (PYF), Guam (GUM), Japan (JPN), Kiribati (KIR), Lao People’s Democratic Republic (LAO), Malaysia (MYS), Marshall Islands (MHL), The Federated States of Micronesia (FSM), Mongolia (MNG), Nauru (NRU), New Caledonia (NCL), New Zealand (NZL), Niue (NIU), Northern Mariana Islands (MKD), Palau (PLW), Papua New Guinea (PNG), Philippines (PHL), Republic of Korea (KOR), Samoa (WSM), Singapore (SGP), Solomon Islands (SLB), Tokelau (TKL), Tonga (TON), Tuvalu (TUV), Vanuatu (VUT), and Viet Nam (VNM), and Wallis and Futuna (WLF). The data were obtained from the World Population Prospects 2022 [8]

Japan is at the forefront of population ageing globally. As Fig. 1 (panel a) shows, the proportion of people aged 65 or above (hereafter, the ageing rate) in Japan has persistently grown over the past 50 years and nearly tripled, reaching 29% of the global population in 2021 [8]. The country has been a global leader in population ageing, with a projected ageing rate of 38% by 2060, primarily due to continuing declines in mortality and fertility rates. Increasing numbers of older people dependent on public pensions, healthcare, and long-term care (LTC) and a declining working-age population endanger the sustainability of labour supply and financing systems. The old-age dependency ratio, defined as the ratio between the 65-or-older and the working-age populations (15–64-year-olds), has increased more drastically than the ageing rate (Fig. 2, panel a). About 50 years ago, this ratio was 1:10—one person aged 65 or over for every 10 of working age’ in 2020, it was approximately 2:1, and it is expected to be 1·3 by 2050. Moreover, due to the ageing of the baby boomers and their children, the proportion of people 75 years and older is also expected to increase in Japan, further increasing LTC demands.

Countries in the WHO’s Western Pacific region, which are widely diverse in terms of their population/geographical size and socioeconomic development, are all either experiencing or are expected to experience rapid population ageing. The old-age dependency ratios by quartile for those countries are shown in Figs. 1 and 2. In 2020, most had ageing rates of around 10% or lower. Their average ageing rates and old-age dependency ratios were approximately 9% and 14%, respectively—equivalent to levels seen in Japan’s rapid economic growth in the late 70 s through early 80 s that saw. Population ageing is expected to continue, at various magnitudes, for all Western Pacific countries, to an average 28% ageing rate and 51% age dependency ratio by 2100.

**Fig. 3:**
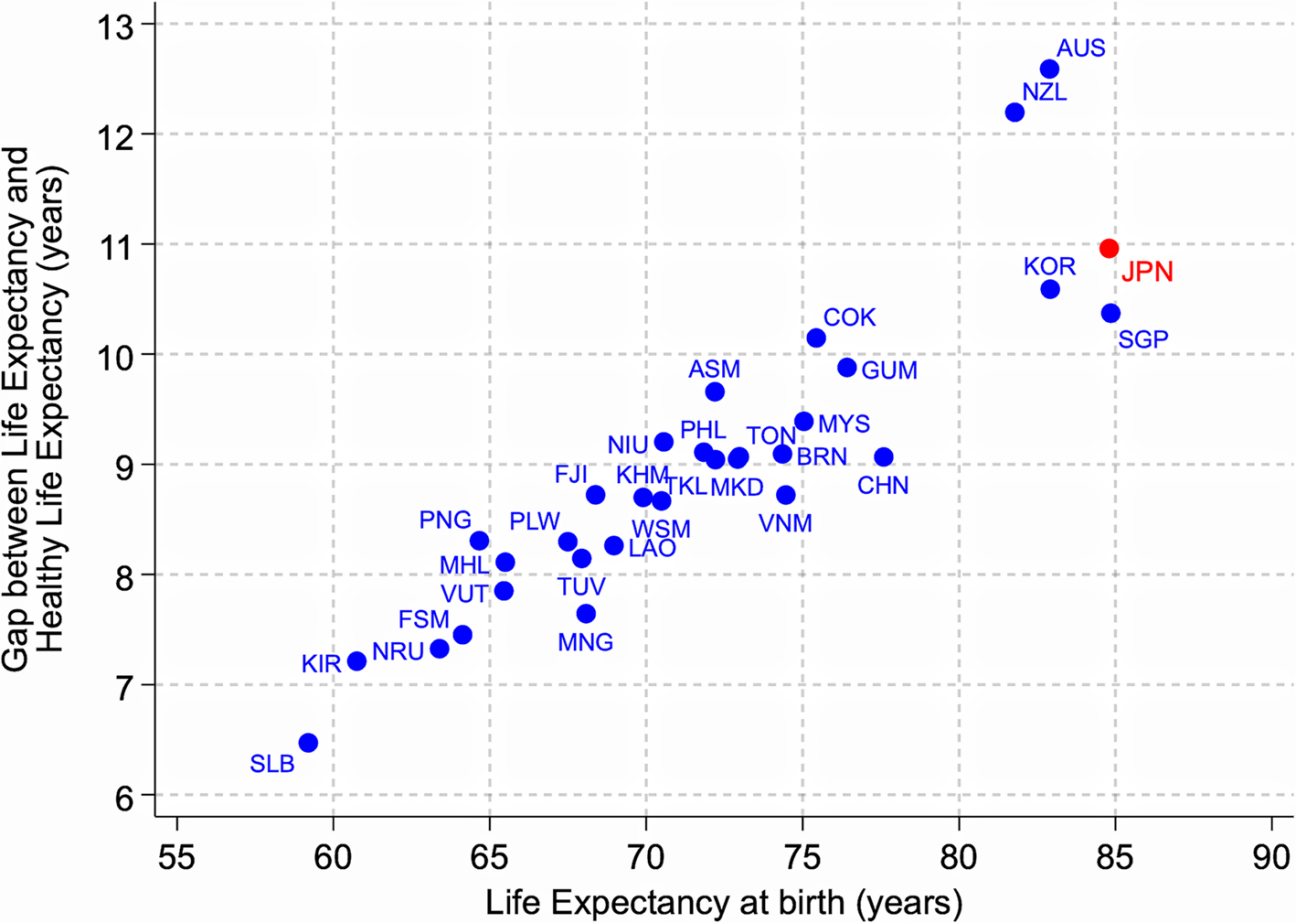
The correlation between life expectancy at birth and the number of years lived with disease or disability, 2019. Note: HALE denotes healthy life expectancy, indicating the average number of years that people in each country can expect to live in full health. The most recent available data for each country were obtained from the Global Burden of Disease Study 2019 [10]. Countries included here are American Samoa (ASM), Australia (AUS), Brunei Darussalam (BRN), Cambodia (KHM), China (CHN), Cook Islands (COK), Fiji (FJI), French Polynesia (PYF), Guam (GUM), Japan (JPN), Kiribati (KIR), Lao People’s Democratic Republic (LAO), Malaysia (MYS), Marshall Islands (MHL), The Federated States of Micronesia (FSM), Mongolia (MNG), Nauru (NRU), New Zealand (NZL), Niue (NIU), Papua New Guinea (PNG), Philippines (PHL), Republic of Korea (KOR), Samoa (WSM), Singapore (SGP), Solomon Islands (SLB), Tokelau (TKL), Tonga (TON), Tuvalu (TUV), Vanuatu (VUT), and Viet Nam (VNM)

Figure 3 presents life expectancy at birth and the gap between life expectancy at birth and healthy life expectancy for countries in the Western Pacific region. The average life expectancy at birth in Japan is about 85 years, with approximately 11 years of life spent living in poor health due to disease and/or injury. Other countries in the region, such as the Republic of Korea, Australia, and New Zealand, face similar or worse situations. Furthermore, people elsewhere, such as Cook Islands, American Samoa, Niue, spend disproportionately longer periods of their lives in poor health (9–10 years) compared to Japan, although their life expectancy is around 10 years shorter. This suggests that older people in all countries and areas need health and LTC services to maintain or improve functional ability and wellbeing because of declining intrinsic capacity [9].

## Rationale for public long-term care insurance

Unlike normal goods and services, which are purchased at the pure private market (e.g. cleaning services and haircuts), many governments intervene in the LTC market via financing by tax and/or social insurance and controlling the price, quantity, and quality of services. Why should the government make these interventions? This is because these interventions make the system more efficient by addressing issues related to imperfect information (i.e. buyers, sellers, or both are not completely certain about the nature of goods and services) and bounded rationality (i.e. choice complexity results in the inability to choose welfare-maximising options) [11]. Issues related to imperfect information are mitigated through the transaction of LTC services at a quasi-market where products’ prices and quality are under government control. In Japan, although many people do not need LTC until the age of 85 [12] (approximately, the average life expectancy), some require it for daily life support. In this sense, insurance-managed LTC is welfare-enhancing. However, actuarial private insurance experiences welfare losses due to imperfect information and bounded rationality due to 1) non-independent probabilities of events (which are known and less than one) across individuals and 2) non-symmetric information: purchasers not knowing the best options to maximise utility and providers facing uncertainty about purchasers’ future LTC needs.

To purchase private insurance, an individual needs to sign a contract with an agency to cover the time from when they first opt for LTC services until their death (a 11-year period, on average in Japan; Fig. 3). In a long-duration contract, actuarial insurance suppliers face inefficiencies due to uncertainty and non-independent probabilities, considering the duration of LTC needed in the future. The nature of future developments is also unknown. For example, medical development may enhance individuals’ health, thereby reducing their dependence on LTC while simultaneously increasing the period of LTC requirement with a longer life expectancy. Similarly, long-duration insurance purchasers experience uncertainty because they do not know the type, duration, and cost of services they may need in the future. Furthermore, individuals’ choices may not be rational due to bounded rationality since choosing the best services requires the possession of professional knowledge of LTC services. Moreover, users may undergo cognitive decline associated with ageing [13]. In addition to these issues, asymmetric information in the principal–agent relationship for voluntary insurance, moral hazard (e.g. over-dependence on LTC services), and adverse selection (e.g. cream-skimming of low-LTC-risk individuals) may also produce welfare losses.

Further, the burden of informal care on family members may produce two additional issues: (1) the inability to access quality care, which leads to deteriorating autonomy and quality of life and (2) the heavy burdens of informal care, such as caregivers’ deteriorating health [14, 15] and opportunity costs (economic losses) [16, 17]. In many countries, the burden of unpaid care work is borne largely by women [18], and this raises gender inequity issues. In this regard, unnecessary costs and pressures on the healthcare sector can be averted by distinguishing healthcare and LTC needs. Therefore, a well-designed universal public LTC insurance scheme can enhance gender equity and the wellbeing of the population and improve system efficiency.

## Pathways to the establishment of a public LTC insurance system in Japan

Until the establishment of the public LTC insurance scheme in 2000, post rapid economic development, Japan spent 30–40 years developing health and welfare systems for the older population prior to experiencing its full-blown population ageing problem. In contrast, some countries in the region may face time and budget constraints in consolidating care systems for older people. In this section, based on documents from the Ministry of Health, Labour, and Welfare in Japan [19–21], we briefly provide an overview of pathways to the establishment of universal LTC insurance to help countries establish their LTC system efficiently.

Health and welfare policies for the older population in Japan have been in development since the *Act on Social Welfare for the Elderly* was established in 1963. Prior to this, the LTC needs of older people were met through informal care provided by their family members or public assistance provided to those living in poverty (i.e. the provision of shelters, etc.). However, the capacity of family members to bear LTC burdens drastically declined. This was due to the changes in improved gender equality, family structures, and living arrangements resulting from industrialisation and the subsequent concentration of population in urban areas during and after the rapid economic growth. Hence, universal LTC services for the entire older population were urgently needed; this resulted in a shift from providing selective care to poor individuals on public assistance.

In the 1960s, after the *Act on Social Welfare for the Elderly* was enacted, the number of individuals benefiting from LTC policies increased through the institutionalisation of home-visit care and care facilities for non-poor individuals. Meanwhile, institutional care was not very accessible because of the limited number of care facilities, which resulted in its use being means-tested and prioritised by individuals in poverty.

In 1973, health and welfare policies for the older population were expanded and improved. Reducing the financial burden of healthcare spending for the public was a matter of policy concern, as many older adults suffered multiple chronic conditions and spent a lot of time and money on treatment, cure, and rehabilitation. For this reason, out-of-pocket payments for people aged 70 or over (65 or over for the bedridden) were waived by the national and local governments. As a consequence, together with the shortage of care facilities, this increased *social hospitalisation*, a phenomenon where older people admitted to a hospital prolonged their stay even though they did not need further medical treatment, as an alternative to long-term facility care; the costs were paid by healthcare insurance. Hospitals were more accessible and affordable than LTC facilities for families of people in need of LTC to reduce their caregiving burden. Despite the similar levels of hospitalisation rates in Japan and in other industrialised countries, the number of hospitalised older people drastically increased between 1963 and 1993, with about half of the hospital beds occupied by older people [22]. The authors note that approximately one-third of those patients were hospitalised for more than a year, suggesting most of the hospitalisations may have been due to social hospitalisation.

To address this issue, planning and laws related to LTC policies were rapidly developed since the late 1980s. Considering that older people need more nursing and personal care to support their daily lives than that provided with the medical treatment at hospitals, skilled nursing facilities for older people, where they could receive the necessary medical care and support for their participation in activities of daily living (ADL), were established. Moreover, to further benefit the older population, in-home care provision was developed by formulating the *Gold Plan*. Due to progress in planning and enacting laws among municipalities and prefectures, better in-home and institutional care environments were established in the 1990s. Nevertheless, these schemes were managed by a safeguarding system, where the government identified the individuals who required LTC and prescribed the services they would need; hence, it did not reflect the demands of the service users. The policy priority from the late 1980s was to expand the LTC services to respond to increasing LTC needs. Together with the development of the LTC system and the increasing demand for meeting user preferences for services, financing LTC was recognised as one of the most important policy issues.

## Functioning of the public LTC insurance in Japan

**Fig. 4:**
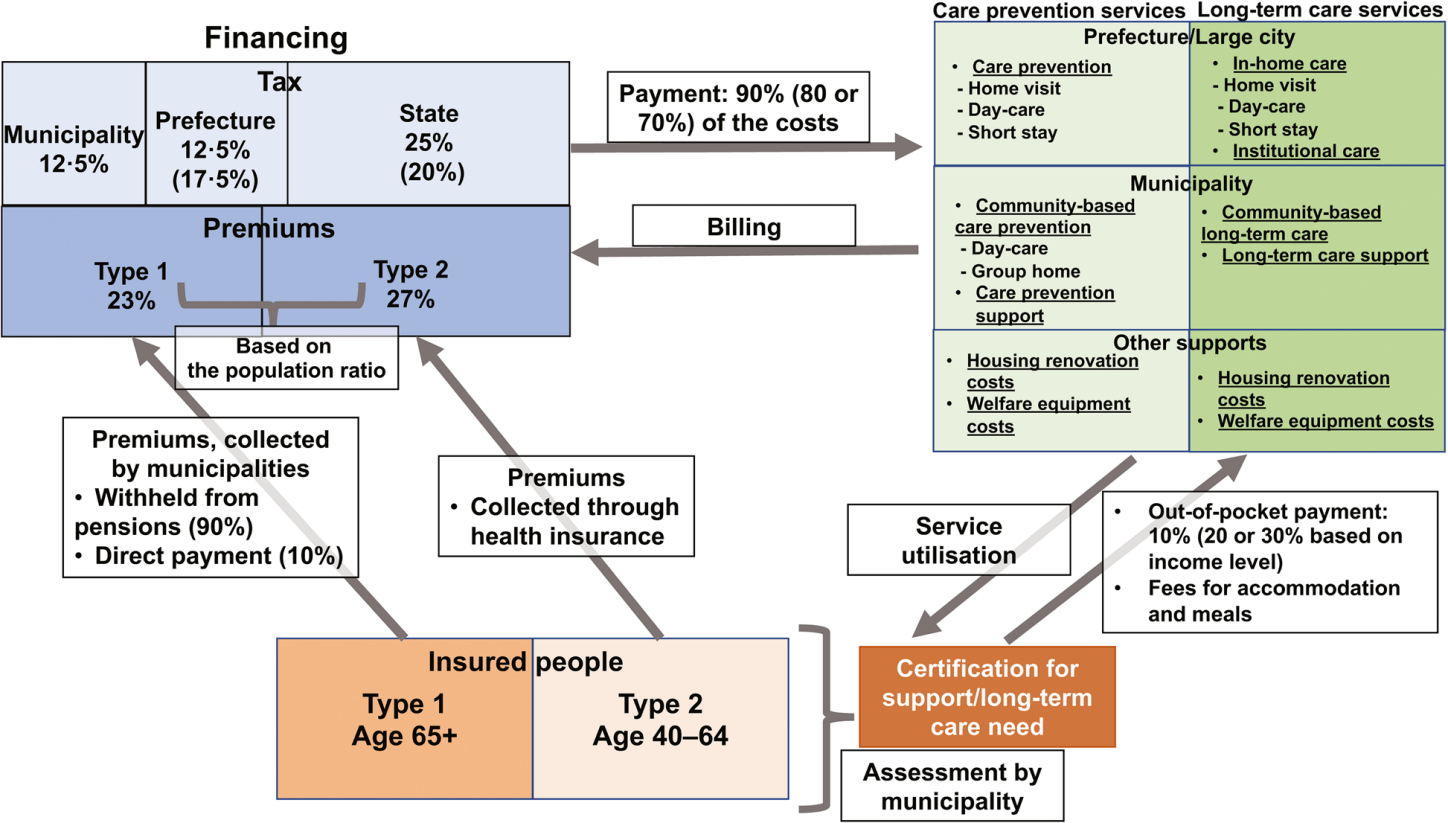
Public long-term care financing system in Japan (as of April 2022). Note: The figure was created by the authors, based on the document published by the Ministry of Health, Labour, and Welfare in Japan [21]. Regarding financing, prefectures and the national government pay 12·5% and 25% of costs for in-home care, respectively, while they pay 17·5% and 20% for institutional care, respectively

Japan implemented the public LTC insurance in 2000 after the enactment of the 1997 *Long-Term Care Insurance Act*. This new policy aimed at supporting the older population’s independence and sharing the cost burden with society through social insurance managed by municipalities. A contract between the LTC services’ users and service providers allowed users to choose healthcare and LTC services based on their needs, with public support and care management techniques. The following sections provide an overview of Japan’s LTC system (Fig. 4), based on publications of the Ministry of Health, Labour, and Welfare [19–21, 23].

### Insured population

Japan’s public LTC insurance system provides insurance to all individuals aged 65 or above (type 1), and public healthcare insurance provides insurance to individuals aged 40–64 (type 2). Different eligibility criteria determine this insurance system’s beneficiaries. Type 1 individuals include those who need support for participating in ADL and LTC (e.g. bedridden individuals, individuals with dementia, etc.). In 2020, approximately 6.69 million type 1 individuals received benefits [12]. Type 2 individuals require LTC due to specific age-related conditions (e.g. young dementia, end-stage cancer, and articular rheumatism). Approximately 0·13 million type 2 individuals received LTC benefits in 2020 [12].

Older people (or their family members) requiring LTC benefits submit an application at their municipality office; their LTC need-level is assessed based on a nationwide uniform standard, which includes an objective test and their attending physician’s evaluation. They are classified into support need-level 1–2 or LTC need-level 1–5, depending on their level of requirement. Individuals in support-need-levels 1–2 can engage in ADL without much support from others; they also receive services to prevent further deterioration in physical and cognitive function. Meanwhile, those in LTC need-levels 1–5 require assistance to engage in ADL. They can avail either in-home or institutional care. In the case of in-home care, a care manager assists in care planning based on the individual’s needs. Significantly, the assessment methods and criteria for judging LTC need-level have not been revised since the last revision in 2009.

### Service coverage

Depending on the severity of individuals’ care needs, LTC insurance offers services related to prevention and care. These are designated and managed by municipalities and prefectures and include in-home care support (e.g. care planning, administrative support for LTC utilisation, and follow-ups), in-home services (e.g. home visits, day-care, and short-stays), and services for institutional care. Municipalities provide community-based support and services. Additionally, financial support is available for renting or purchasing assistive devices (e.g. wheelchair and bathing aids) and for making home renovations to adopt a barrier-free design. Japan’s LTC insurance system also covers preventive care, such as ADL support and rehabilitation, to help older adults avert further health deterioration. The preventative care programme, which is mostly conducted by municipalities, has been providing effective and efficient community-based support since 2017 by incorporating various services into the immediate environments of various individuals and organisations. Before this programme was implemented, services aimed at preventing disability primarily focused on functional training. They paid little attention to older adults’ living environment and social engagement, which can encourage continuous participation in activities that support physical and mental health. Therefore, the government proposed a preventative-care service policy integrating functional training and approaches to address the social determinants of health (e.g. older adults’ living environment and social activities).

In Japan’s public LTC insurance system, through the partial adoption of the market mechanism (i.e. a quasi-market), users select LTC services based on their preferences, which are then provided by public and private organisations following government regulations. Therefore, individuals’ service choices and government regulations, both control the quality of services. A care manager creates a care plan for each individual and plays a vital role in the decision-making regarding LTC services. As entry barriers, institutional care service providers are restricted to public or non-profit private organisations (e.g. medical institutions and social welfare corporations). In contrast, profit organisations can provide in-home care services, including in private residential homes and serviced housing, for older people. For the quality control of LTC services provided under the public insurance scheme, LTC workers are required to have certain qualifications, training, and experience. Moreover, service providers receive approval from a prefecture or municipality based on certain criteria such as having sufficient qualified workers, necessary equipment, and a proper operating system required for a facility.

### Integrated continuum care in the community

The national government encourages local governments and individuals to utilise in-home care instead of institutional care owing to increased LTC costs and LTC facilities’ shortage. Governments have developed *the community-based integrated care system*, with *community general support centres* to enable this [23, 24]. These centres provide LTC-related medical care and services. They cater to public health and welfare, perform general consultations and advocacy-related tasks, and provide comprehensive and continuous support for care management and prevention. These services are provided by interprofessional teams comprising public health nurses, certified social workers, and senior-level LTC support specialists. The centres were established by municipalities in each area of Japan, covering 20–30 thousand citizens (this usually corresponds to junior high school districts); some of them are managed by non-profit (e.g. social welfare corporations) and private organisations.

### Financing

Starting from approximately JPY 3·6 trillion (~ USD 28·4 billion, equivalent to 0·7% of GDP) in 2000, LTC costs have increased rapidly, owing to expanded services and an increased number of users, reaching approximately JPY 10·8 trillion (or ~ USD 84·4 billion, equivalent to 2% of GDP) in 2020 [25]. Public LTC insurance cost is financed via three sources: out-of-pocket payments by users, public expenditure, and insurance premiums. Service users bear LTC service fees at a 10% co-payment rate (20% or 30% for those above a certain income level), excluding the costs for meal and accommodation costs, and other non-LTC-related expenses. The remaining costs are equally shared by the public and insurance premiums.

In the context of public finance, municipalities, prefectures, and the national government share the costs, depending on the type of care (i.e. in-home or institutional care) provided. Insurance premiums for type 1 individuals vary across municipalities and are determined by individuals’ income levels. To contain the costs, caps for each level of support and LTC need category are in place, and the user bears any cost above the cap. The costs borne by users and the fees for utilising the services are nearly identical nationwide, with small regional variations. For instance, per capita monthly LTC costs in April 2021 (the sum of out-of-pocket payments, insurance benefits, and public expenses) were approximately JPY 28,000 (~ USD 230) and JPY 202,000 (~ USD 1,680) for individuals receiving disability prevention and LTC benefits, respectively.

Furthermore, the public LTC insurance system is reviewed every three years to enhance its sustainability (e.g. re-examining the fees for services, insurance premiums, and levels of benefits) and function while considering factors such as LTC service utilisation, the balance of payments of service providers, and demographic factors. For instance, the latest reform in 2021, included four basic policies [26]: 1) strengthening the system’s resilience against the pandemic (e.g. creating infection control guidelines for care providers); 2) furthering the community-based integrated care system (e.g. adding credits or guidelines for dementia care, terminal care, home-visit care, integrated health, long-term care and matching services to regional characteristics); 3) furthering assistance for self-help and prevention to limit increasing severity of conditions (e.g. rehabilitation/functional training, oral and nutritional care, quality assessment of care, and evidence-based LTC); 4) improving LTC staffing and enhancing workplace environments (e.g. improvement of working conditions, utilisation of technologies, reduction of administrative tasks); and 5) enhancing system stability and sustainability (e.g. revising service fees).

### Financial protection

In addition to enabling low levels of out-of-pocket payments, the government equips the LTC system with financial protection from catastrophic LTC spending and adequate financial support for low-income individuals in many ways.

First, multi-stage insurance premiums determine individuals’ premium fees based on their income level. As a standard, insurance premiums comprise nine levels, although some municipalities set additional levels to reflect area-specific income distribution. Second, regarding out-of-pocket payments, while the co-payment rate for individuals aged 40–64 is 10%, the rate for those aged 65 or over is determined by the individual’s ability to pay. The standard rate is 10%, but it may be 20% or 30% for higher income levels. Third, the combined healthcare and LTC costs for some individuals can become excessive (e.g. multiple household members with LTC needs). This burden is mitigated through reimbursements up to a certain ceiling amount, which is determined by household members’ age and income level. Fourth, low-income users using institutional care can receive financial support from public bodies to cover meal and accommodation costs at institutional care homes. Fifth, individuals receiving public assistance can use LTC services without the financial burden of both insurance premiums and out-of-pocket payments. However, in some cases, overuse of services due to the absence of out-of-pocket payment burden can be of concern. Finally, to protect individuals with borderline poverty status from impoverishing LTC expenditure, financial support is provided through reductions in insurance premiums and out-of-pocket payments.

In summary, Japan’s public LTC insurance system provides comprehensive service coverage for the older population’s health and welfare through a quasi-market. It provides financial protection from catastrophic expenditure and financial support to low-income individuals, and its finances are managed mainly through social insurance.

## Challenges and prospects of Japan’s LTC system

### Financing

The number of people requiring LTC in Japan has increased from 2.18 million in 2000 to 6.69 million in 2020 [12]. With an expected increase in the number of people aged 75 or above, LTC costs are also expected to increase continuously [27]. Moreover, insurance premiums have increased based on reviews of the LTC system. Currently, the average LTC premium for type 1 individuals is JPY 6,014 (~ USD 50) in 2021–2024 [28]. Considering LTC facilities’ shortage, the government plans to shift its focus from institutional care to in-home care.

### Labour force shortage

#### Future prospects of LTC workers

According to Japan’s Ministry of Health, Labour, and Welfare [29], the required number of LTC workers will increase from 2.11 million in 2019 to 2.43 million in 2025 and 2.80 million in 2040. Although the rate of increase in the number of people aged 75 or above after 2025 is expected to slow down, the supply of LTC workers will remain a challenge due to the retirement of baby boomers’ children around 2040. To resolve this expected shortage, improvements in working conditions and wages are imperative, especially considering workers’ physical and mental burdens. If the working conditions and wages are not improved, attracting and retaining individuals in the LTC workforce will be difficult. However, LTC workers’ wages are related to the fee for services, which means that raising wages may lead to an increase in insurance premiums and/or tax. Further, workers must also be offered secure long-term opportunities for employment and proper career training (e.g. qualifications and task shifting or sharing among healthcare and LTC workers).

#### Utilisation of technologies

The adoption of new technologies (e.g. information and communication technology and nursing care robots) will reduce the time spent on administrative work, expedite the process of user identification, increase workers’ productivity, and decrease job-related physical and mental burdens. Robotic technologies are expected to assist caregivers in providing care related to body transfer, excretion, monitoring, communication, and bathing [30]. These technologies include wearable power assisting suits, automated excretion devices, electric-powered walking trolleys, and monitoring sensors. However, challenges related to issues such as cost, user-friendliness, communication of the advantages and disadvantages of such technologies, and distrust in robot-based care must be overcome.

Further, information and communication technology can be used to reduce LTC workers’ administrative workload, thereby enhancing operational efficiency, as service quality may be well controlled and improved when technologies are utilised to monitor the services provided and evaluate the data. For example, analysing user characteristics and the care plans suggested by care managers may lead to easier and more accurate care planning. Although Japan’s LTC insurance system aims to respond to the needs of older people and provide support for ADL to help them retain their dignity and autonomy, the services provided are not based on scientific evidence. Thus, to enhance the quality and efficiency of care and prevent further health deterioration, the Ministry of Health, Labour, and Welfare put forward the *Long-term care Information system For Evidence* (LIFE) [31]. This scheme calls for collecting and analysing scientifically-validated quantitative and qualitative data about system users’ characteristics and health statuses and the types of care provided so that the evidence-based plan-do-check-act cycle of care can be adopted to enhance LTC services’ quality and provide effective preventive services that avert further health deterioration.

#### Foreign LTC workers

Despite LTC workers’ shortage, the Ministry of Health, Labour, and Welfare initially did not accept immigrant workers because it may cause difficulties in ensuring good quality services under the public LTC insurance system, considering immigrants’ barriers related to language and qualification requirements. However, the government has recently started accepting foreign LTC workers from various countries in Southeast Asia through four channels [32]: (1) the Economic Partnership Agreement, (2) the status of residence for nursing care, (3) the *Technical Intern Training Program* and (4) *Specified Skilled Worker*. Nevertheless, heavy reliance on foreign workforces may not be sustainable or ethical because population ageing is a global concern, resulting in increasing the LTC needs of foreign countries.

### Support for people with dementia

Along with an increase in the number of people aged 75 or above, the number of people with dementia may increase owing to its higher prevalence rate among older adults [33], although ageing may not automatically increase its prevalence due to improvements in its risk factors (e.g. health behaviours, the treatment and control of non-communicable diseases, and social activities) [34]. Considering the enormous social costs of dementia [35], along with other issues, such as an increased number of road traffic accidents involving people with dementia as drivers or pedestrians, provision of support for financial asset management, protection of consumers with cognitive decline from becoming victims of fraud and other scams, the provision of support to prevent dementia and to people living with dementia is indispensable. To manage these issues, the government has developed a law, along with planning recommendations and policy principles related to dementia, to delay the onset and progress of dementia and enhance the lives of people with dementia and their families. These schemes include five pillars [36]: 1) enlightenment about dementia; 2) prevention; 3) provision of health and long-term care and support for caregivers; 4) furtherance of barrier-free design and the protection of people with dementia from frauds, community-based care, support for people with young dementia, and social engagement of people with dementia; and 5) research and development, industrial promotion, and their international expansion. The development of supporting people with dementia is still in progress, and each municipality is designing systems that reflect the municipality’s situation regarding available resources for dementia care.

### Integrated continuum of healthcare and LTC

According to estimates by the National Institute of Population and Social Security Research [37], among households with the head of the household aged 65 or over, the proportion of single-person households is expected to increase from 32·6% in 2015 to 40·0% in 2040. This implies that the number of older people who cannot rely on their families will increase, thereby increasing the importance of the support that they may receive from public or private sectors in the community. With the furtherance of in-home care, the integration of healthcare and LTC in the community is vital to respond to the needs of older people, who often have multiple chronic conditions. Given the separate insurance systems for healthcare and LTC, as well as insufficient interprofessional collaboration and home-visit medical care, the integration of healthcare and LTC service provision must also be improved. The Ministry of Health, Labour, and Welfare promotes this integration through each municipality’s initiative. Eight necessary items have been indicated in this regard [38]: 1) understanding of healthcare and LTC resources in each region and sharing of resources among stakeholders; 2) understanding and consideration of challenges in the integration of in-home medical care and LTC and related countermeasures; 3) promotion of the system’s establishment for continuous medical and long-term care provision; 4) support for information sharing between health and LTC workers; 5) consultation for the integration of healthcare and LTC (e.g. allocation of coordinators); 6) training for healthcare and LTC workers; 7) enlightenment of citizens; and 8) regional coordination across municipalities. Each municipality is expected to design a system by considering healthcare and LTC resources and other issues that could be unique to the region (e.g. geographic factors and citizens’ demands).

### Services outside the purview of insurance coverage

With the declining supply of LTC workers and increasing varieties of LTC services required by older people, it may not be plausible to support every individual receiving in-home care solely through public LTC insurance benefits. Hence, private market services, which are outside the insurance coverage’s purview, may play an important role. Three ministries in Japan jointly published a guidebook about these types of services [39], indicating that successful cases may be equipped with interpersonal communication and social activities (e.g. hobbies and group activities provided with care), services related to domestic work (e.g. cleaning and accompanied shopping), or activities that a person could engage in without any difficulties before needing care (e.g. travels accompanied by helpers). Nevertheless, as mentioned earlier, it is difficult for users to choose the necessary services themselves, and an overuse of these services may discourage the older population from living independently. Therefore, these services, if expanded, need to be well incorporated within the current public LTC insurance system to supplementally satisfy the demands of older people not covered by the formal LTC services with the essential care provided by the public LTC system.

## Implications from Japan’s experience

We provided an overview of Japan’s public LTC insurance system to help other countries that are experiencing (or are expected to experience) problems associated with population ageing. The two important elements of universal health coverage—(1) enabling everyone to access the necessary health services and (2) providing financial protection—are vital for not only healthcare but also LTC in the context of population ageing.

Even with large heterogeneities across countries in size, socioeconomic development, and ageing rate, Japan’s experience with the LTC system amidst current challenges has important implications for countries planning to implement an LTC insurance system. This is because the LTC system has certain common key issues (e.g. financing and the quality and quantity of service). As mentioned earlier, well-designed social insurance for LTC is welfare-enhancing and helps avoid potential market failure. Therefore, countries adopting the LTC system can benefit from a combination of public financing and service provision through the quasi-market mechanism. Moreover, population ageing is a major challenge for the LTC insurance system, particularly in terms of financing and labour force shortage. Under Japan’s system, people aged 40 or over are enrolled for insurance and pay insurance premiums based on their income level. Together with the premiums, the LTC insurance system is financed by tax and users’ out-of-pocket payments. To enhance financial sustainability, the government reviews the system triennially, adjusting insurance premium rates, benefits, and the fee for services, as required. From Japan’s experience, four main ways are feasible to address financial challenges: (1) by reducing the number of care recipients by health improvement, (2) by increasing the number and/or productivity of care workers by improving work environments and utilising technologies, (3) by increasing the LTC insurance’s revenue by raising taxes, premiums, and/or out-of-pocket payments, and (4) by revising the fee for services. Additionally, regular careful reviews and revisions of the system will be beneficial to enhancing financial sustainability and responding to unexpected situations (e.g. a pandemic).

The types of LTC services to be offered and their quality should be carefully determined to support users’ wellbeing as well as that of their family members. It should reflect users’ preferences so that they can live their lives in their own ways. The LTC services covered by Japan’s public LTC insurance benefits are provided by public or private providers under the quasi-market mechanism, which partly contributes to maintaining and improving services’ quality. In partial compliance with the guidelines for integrated care for older people [9], these cover comprehensive healthcare, welfare benefits, and support for participating in ADL, as well as preventive care. Moreover, the integrated continuum of healthcare and LTC is important. In home-based care, where different workers (e.g. medical and LTC workers) provide healthcare and LTC services, interprofessional coordination may not work well. Therefore, the care system in each system must involve care coordinators or other professionals and consider available healthcare and LTC resources as well as other related factors to comprehensively address citizens’ welfare, health, and LTC needs.

To achieve universal health coverage, the LTC system must provide financial protection from catastrophic spending and adequate financial support to low-income individuals. In this regard, the Japanese system offers insurance premiums, out-of-pocket payments, a ceiling for combined healthcare and LTC spending, financial support for services not covered by the insurance benefits, and public assistance. Other countries could also incorporate these types of income protection into their systems to avert impoverishing spending and foregone care, which may result in the deterioration of the health and welfare of older people and their family members.

In addition to these issues, it should be noted that even without severe functional disabilities, the physical and cognitive functioning of many individuals may decline as a part of normal ageing [40]. Together with the increase in the proportion of single-person households, the furtherance of in-home care will reinforce the need for services outside the purview of insurance coverage and age-friendly environments to help older people live autonomously. In our daily lives, we engage in not only ADLs (i.e. bathing, clothing, and toileting) but also instrumental ADLs (e.g. shopping for various goods/services, telephone call, texting, looking after a pet, cleaning a garden, etc.), which the public LTC insurance does not cover. In this regard, older people can purchase goods and services in the private market in the same way as younger people. However, companies may prey on older people purchasing private market goods and services or exclude them from the purchase due to their declined cognitive functioning. Companies may hesitate to sell products to avoid unnecessary troubles. Some companies may even try to abuse their characteristics, such as lower consistency in risk perception, resistance to framing, inaccurate use of decision rules in choices with multiple attributes, overconfidence in their knowledge, and higher resistance to sunk costs [41]. Therefore, consumer protection and customer-first business practices are important to help older people safely engage in economic activities.

Additionally, ensuring access to necessary care is essential as an element of UHC. With a potential decline in intrinsic capacity, older people will need comprehensive care to maintain their functional abilities and wellbeing [9]. However, they may experience unmet needs for care due to several reasons. First, regional differences in health behaviours and resources for care can affect one’s access to care [42–44]. Hence, appropriate allocation of healthcare resources is needed to reduce disparities in the availability of services. Moreover, community-based integrated care requires further development to utilise limited resources efficiently. Second, help-seeking may not be easy for those living alone or suffering from social isolation due to a lack of family/social support, care avoidance, withdrawal, resignation, and low expectations [45]. Progressive cognitive decline can also hinder people from detecting their issues. Under Japan’s LTC system, eligibility for LTC service utilisation is judged from an application made by themselves or their family members. However, this process may cause difficulty for people with cognitive decline in applying for it. Therefore, it is essential to detect people in need and monitor their status to provide adequate management and care [9]. Third, people living under adverse socioeconomic circumstances may be unable or hesitate to access necessary care through various channels. These channels include low health literacy, financial hardships, heterogeneous preferences (e.g., high time preference rate), and poor health status restricting access [46–48]. Financial protection for those with financial hardships, education for improving health literacy and help-seeking are essential to address socioeconomic inequality in access to care.

Further, our lives revolve around advanced technologies that require complex decision-making (e.g. cashless payment, financial asset management, use of public transportation, walking around maze-like stations, department stores, and cities). Many of these technologies are convenient for younger people whose cognitive and/or physical functioning is healthy, but they may be inconvenient for older people. Therefore, an age-friendly city’s requirements [49] must be re-recognised, and this idea must be incorporated into various aspects of our living environment. In Japan, the Tokyo Metropolitan Government and government agencies develop guidelines regarding the protection of older people as consumers, utilising implications of financial gerontological studies that integrate research on ageing with finance and business concerns, while simultaneously considering older peoples’ behavioural characteristics [50–52] in addition to their physical and mental health. However, these guidelines, their application, and dissemination must be further developed. To enable older people to live a healthy and productive life, their health requirements, as well as requirements in other aspects of life, must be considered. In this regard, the involvement of multisectoral corporations involved with all stakeholders, including governments, public and private sectors, professional workers (e.g. health professionals and lawyers), academia, non-profit organisations, and citizens, is essential to building an age-friendly society that addresses the various needs of older people.

To conclude, Japan’s approach to LTC may not be universally generalisable given the differences in population/geographical size, socioeconomic development, population ageing, and culture across countries. However, the challenges faced by older people may be common. Moreover, some common issues affect LTC provision, including market failure, financing, and service production. Municipalities within Japan are largely heterogeneous in terms of size, socioeconomic status, resources for healthcare and LTC, and population ageing rate. Under the common public LTC insurance system, each municipality and prefecture designs and implements the system based on its LTC situation. While we do not discuss this in detail, countries with features common to Japan’s municipalities (e.g. the size and available resources) may obtain implications from the successful cases of these municipalities. The ultimate goal is for countries to share related insights, enjoy universal health coverage, and achieve healthy ageing.

## Data Availability

Data used in Figs. 1 and 2 are obtained from United Nations. World population prospects 2022. Data used in Fig. 3 are obtained from Global Burden of Disease Collaborative Network. Global Burden of Disease Study 2019 Results.

## Abbreviations

WHO: World Health Organization
LTC: Long-term care
ADL: Activities of daily living
